# A Lab-on-a-Chip Based Automatic Platform for Continuous Nitrites Sensing in Aquaculture

**DOI:** 10.3390/s22020444

**Published:** 2022-01-07

**Authors:** Alexandro Catini, Rosamaria Capuano, Giuseppe Tancredi, Giulio Dionisi, Davide Di Giuseppe, Joanna Filippi, Eugenio Martinelli, Corrado Di Natale

**Affiliations:** Department of Electronic Engineering, University of Rome Tor Vergata, Via del Politecnico 1, 00133 Rome, Italy; catini@ing.uniroma2.it (A.C.); capuano@ing.uniroma2.it (R.C.); g.tancredi@cloudwise.it (G.T.); Giulio.Dionisi@uniroma2.it (G.D.); di.giuseppe@ing.uniroma2.it (D.D.G.); filippi@ing.uniroma2.it (J.F.); martinelli@ing.uniroma2.it (E.M.)

**Keywords:** nitrites, lab-on-a-chip, colorimetry

## Abstract

In aquaculture, the density of fish stock, use of feeding, and surrounding environmental conditions can easily result in an excessive concentration of harmful compounds that require continuous monitoring. Chemical sensors are available for most of these compounds, however, operative conditions and continuous monitoring in water make the development of sensors suitable for long and unattended deployments difficult. A possible solution is the development of engineered automatic labs where the uptake of sample and the contact with water is reduced and the use of a minimal quantity of reagents enables the implementation of reliable chemical assays. In this paper, a platform for automatic chemical assays is presented. The concept is demonstrated with the detection of nitrites based on the well-known colorimetric Griess reaction. The platform is centered around a lab-on-a-chip where reagents and water samples are mixed. The color of the reaction product is measured with low-cost optoelectronic components. Results show the feasibility of the approach with a minimum detectable concentration of about 0.1 mg/L which is below the tolerance level for aquaculture farms.

## 1. Introduction

The concept of precision agriculture and farming is a significant field of research in the ICT community. The implementation of electronic sensors vastly increases the capability to monitor and control the physical and chemical parameters of plants or animal stocks [1,2]. The introduction of electronic technology results in a paradigm shift that enables the change of management of farms with the consequent increase of production quality and quantity and the reduction of the environmental impact through optimization of resources used in the farming process.

Aquatic animals and plants are basic elements of human diets. It is interesting to note that while terrestrial animal husbandry has existed since the beginning of human development, farming of fish and aquatic plants only recently began [3]. The aquatic environment is characterized by features that hinder the possibility of a simple transfer of sensing technologies from terrestrial farming to aquaculture. It is important to consider that water besides being a conductive medium, is a harsh environment because of the corrosion due to dissolved ions and biofouling [4].

Specific issues about aquaculture monitoring require sensor systems suitable to be operated with the same performance for a long period and in varying environmental conditions. In summary, sensors for aquaculture applications should be characterized by low maintenance, low battery consumption, waterproof, unaffected by biofouling and not interfering with the farmed organisms or the environment.

A correct appraisal of aquaculture farm status requires the monitoring of a number of compounds and nitrite ions are among them [5]. Accumulation of nitrites may be attributed to a number of different causes. Nitrogen compounds are natural metabolites, and the abundant use of proteinaceous feed and high density of fish are the main causes of excess nitrite concentration [6]. Additional sources, in particular in freshwater farms, include the overconsumption of fertilizers in the surrounding fields. In aquaculture systems, the normal concentration of nitrites is usually below 1 mg/L. Above 20 mg/L nitrites are toxic for fishes and crustaceans [7].

Given the importance of monitoring nitrites, several detection techniques have been proposed for this scope [8]. Electrochemical [9] and fluorescent [10] sensors have shown sufficient sensitivity for environmental control. However, since these sensors work in contact with water, they are not immune from biological and chemical contaminations that limit their long-time operation.

The subject of nitrites sensors have been covered by several authors; recent review papers offer thorough comparisons between different sensor technologies and methodological approaches [11,12]

In this paper, we report the design, manufacturing and test of an automated platform for colorimetric assays. The core of the developed sensor platform is a plastic lab-on-a-chip device that allows for the mixing of reagents and samples. The use of microfluidic systems is an emerging technology for the development of automatized analytical assays [13].

The functionality of the platform has been tested implementing a commercial kit for nitrites based on the Griess reaction and aimed at con-trolling nitrites in fresh and marine waters.The griess assay is based on the quantitative conversion of Sulfanilic acid to a diazonium salt by reaction with nitrite in acid solution. The diazonium salt is then coupled to N-(1-naphthyl) ethylenediamine, forming an azo dye whose concentration is spectrophotometrically appraised based on its absorbance at 548 nm [14,15]. The Griess reaction can be extended to the detection of nitrates using additional reagents, such as Vanadium(III), to reduce nitrates to nitrites [16].

The development of microfabrication technologies prompted the design of systems t for the miniaturization of on-line analytical chemistry techniques [17]. The cores of these systems, subbed as lab-on-chip, is the combination of microfluidic circuits, for handling minute quantities of samples and reagent [18], and sensors to detect and quantify the reaction products [19]. 

The integration of Griess reaction with a microfluidic system has been previously demonstrated to detect nitrites in oceans [20,21] and in natural waters [22]. The immobilization of Griess reagents in microfluidic channels have been used to develop disposable devices [23]. The selectivity of Griess assay can be limited in complex food samples or biological fluids characterized by the presence of antioxidants and pigments [24]. In aquaculture farms them main concurrent compounds are nitrates and phosphates. The sensitivity of Griess assay to nitrites respect to nitrates and phophates has been found, in a paper immobilized reaction larger than 80 times [25]

In this assay, we have been interested to a system suitable for automatic repeated measurements and thus microfluidic device assisted the continuous uptake of sample, mixing with reagents and discharge with a minimal use of reagent per measurement. The detection of color changes has been performed firstly with a miniaturized spectrophotometer and then with a simple and cheap integrated optoelectronic setup. Results show that even without a dedicated optical design, the minimum detectable concentration is smaller than the limits allowed for aquaculture farms are reached.

## 2. Materials and Methods

### 2.1. Reagents and Chemicals

A commercial, Griess reagents based colorimetric test (sera Nitrite test, Sera GMBH, Heinsberg, Germany) was used in the platform. Griess reagents solution can be prepared following different recipes. Sulfanilic acid is typically dissolved in an acidic solution (e.g., citric acid) and N-(1-naphthyl)ethylenediamine dihydrochloride is used to promote the formation of the colored azo dye. [25]. However, the exact formulation of the commercial reagent used in this paper is unknown except for the presence of hydrogen chloride in N-(1-naphthyl)ethylenediamine dihydrochloride which is reported for safety issues. The reagents mixing occurs without any magnetic stirrers but inside the microfluidic chip. 

Calibration solutions were made from nitrate stock solution (10 mg/L) prepared from sodium nitrate (Sigma Aldrich, 99% purity), smaller concentrations were obtained by dilution with water.

### 2.2. Lab-on-A-Chip Production

The Lab-on-Chip device was designed and manufactured at the University of Rome Tor Vergata. The chip, designed with CAD software (Autodesk Inventor^®^), consisted of several layers of poly (methyl methacrylate) (PMMA) (Clarex^®^, Nitto Jushi Kogyo Co. Ltd., JP; supplied by Weatherall Ltd., Aylesbury UK). Each layer was carved by a computer controlled laser cutting (Trotec Speedy 100, Trotec Laser Inc., Marchtrenk Austria) and then assembled. A polyester release liner was used during the laser cutting to avoid material damages and to maintain transparency. After cutting, the release liner was removed and the layers were piled up to form the whole chip. Layers were glued using temperature cured absolute ethanol (%w > 99.95%w, Sigma-Aldrich, UK). Layers were sprayed with ethanol and then quickly assembled in a custom aluminum holder pre-heated at 70 °C [26]. During the operation, the chip was kept upside-down to favor the leakage from the holes of the excess of non-vaporized ethanol. The holder was placed between custom-made hot plates kept at 70 °C and pressed with a hydraulic press (BETA 3027 10, Sovico Italy) at 100 bar for 10 min. The chip was let to cool at room temperature to prevent cracks around the channels and holes. Finally, a few microliters of glue (COLLACRYL K90, Plastidite SpA, S. Dorligo della Valle, Italy) were pipetted between the layers to promote a stronger adhesion and to avoid any undesirable leakage during the chip operativity. Fluid inlet and outlet were provided by FESTO PUN-3 mm PU tubing and FESTO QSM-M5-3 connectors.

### 2.3. Hardware Design

The spectra of the Griess reaction product were measured with an integrated spectrophotometer (C12666MA, Hamamatsu, Japan). The device is characterized by a spectral response range from 340 nm to 780 nm with a spectral resolution smaller than 15 nm. The maximum spectral response is around 500 nm suitable for the detection of Griess reaction products. The spectrophotometer was integrated into the Arduino shield endowed with a white LED as the light source for color measurement.

The color of the reaction products was measured with an RGB sensor (TCS34725, AMS Premstaetten Austria) endowed with an IR filter to limit the sensitivity to the visible range and a white led as a light source.

Reagents and water input were controlled by piezoelectric minipumps (MP6-Hybrid, Bartels Microtechnik GMBH, Dortmund Germany). In these pumps, the material in contact with the media is polyphenylsulfone that guarantees optimal resistance to aggressive agents. Pumps were primed with Bartels mp6-OEM controller designed for the integration of the pump into a small space. The frequency and the amplitude of the controller were adjusted to achieve the desired flow rate.

As a control unit, the Arduino Nano V3.0 board was used. Arduino Nano is an open-source electronics prototyping platform, based on V3.0 on the ATmega328, flexible hardware and software.

## 3. Results and Discussion

### 3.1. System Design

**Figure 1:**
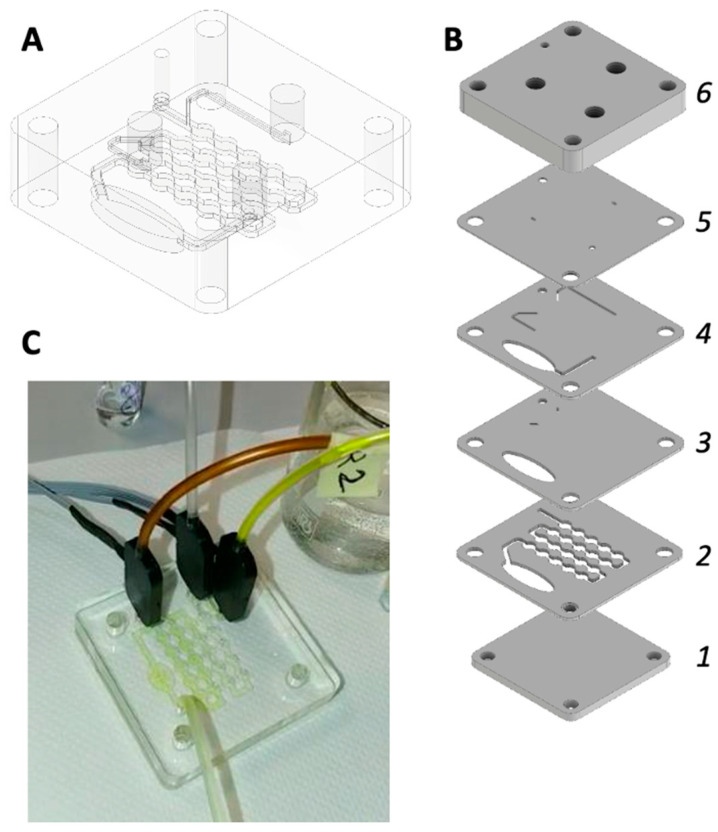
Microfluidic chip: (**A**): Assembly view. (**B**) Exploded view: 1: bottom lid (thickness: 3 mm); 2: mixer and measurement well (thickness: 1 mm); 3: spacer (thickness: 1 mm); 4: reagents channels (thickness: 1 mm); 5: spacer (thickness: 0.5 mm); 6: top lid (thickness: 8 mm). (**C**): picture of the chip connected to reagents and sample inlet and wastes outlet.

Figure 1 shows the construction steps of the PMMA-based Lab-on-Chip. Figure 1A,B show the assembled and exploded design of the chip. The chip is made of six layers. The first layer is the bottom lid, the second contains the mixer channel where sample and Griess reagents mixThe measurement consists in adding 40 µL of each Griess reaction reagents to 1 mL of water sample. The blend of these compounds occurs in the mixer section of the lab-on-a-chip which is designed as a meandered channel made of a sequence of large and narrow sections. The uneven section was found optimal to avoid the formation of air bubbles and to achieve a complete mix of reagents and samples. Air bubbles greatly affect the optical read-out of the reaction products. Bubbles deviate the optical path, reducing the intensity of light, and due to refraction may also alter the color measurement. All these effects impair the estimation of nitrite concentration. The second layer also contains a chamber sufficiently large to allow for the measure of the color of the solution. To avoid the use of expensive and power consuming control micro-electro valves, a third layer was a spacer introduced to prevent the backflow of the sample towards the reagents’ input channels. The fourth layer is the reagents channels, the fifth layer is another spacer introduced as additional protection against the backflow of the solution. Finally, the last layer is the top lid with the orifices for with (8 mm) for the insertion of couplings that allows the inlet of sample and reagents and the outlet of the waste.

Figure 1C shows the assembled chip attached to the reagents, water sample, and waste tubes. The total flow rate of liquids in the chip is maintained at 2.4 mL/min.

### 3.2. Spectrophotometric Calibration

**Figure 2:**
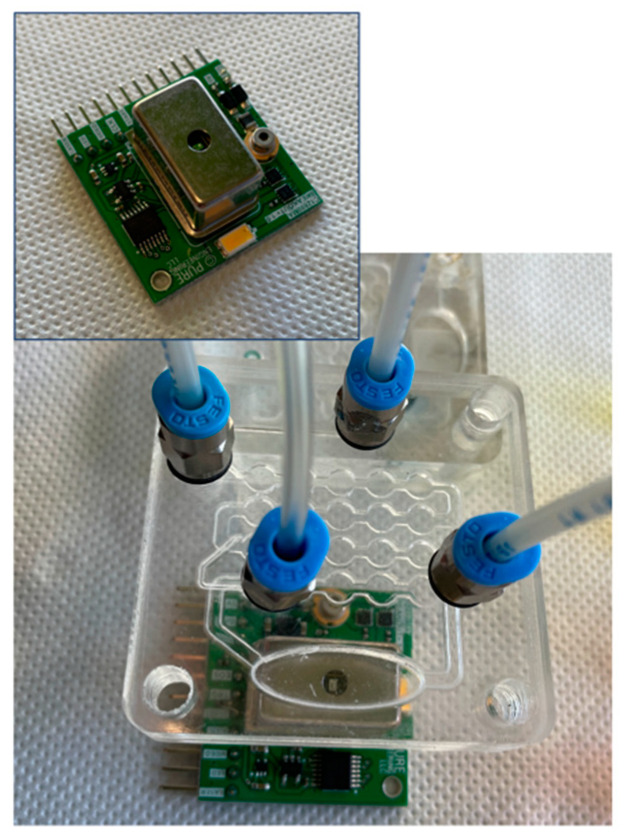
Picture of the microfluidic chip and spectrophotometer. The input slit of the spectrophotometer is in contact with the portion of the chip immediately above the measurement well. A close view of the C12666MA spectrophotometer is shown in the inset.

In the first implementation of the sensor, a spectrophotometer was used to investigate the detectability of changes of color from the measurement well in the microfluidic chip. For the scope, a microspectrophotometer was simply leaned on the surface of the chip. The illumination was provided by a white led embedded in the spectrophotometer breakout board. Figure 2 shows the detail of the spectrophotometer and the measurement arrangement.

**Figure 3:**
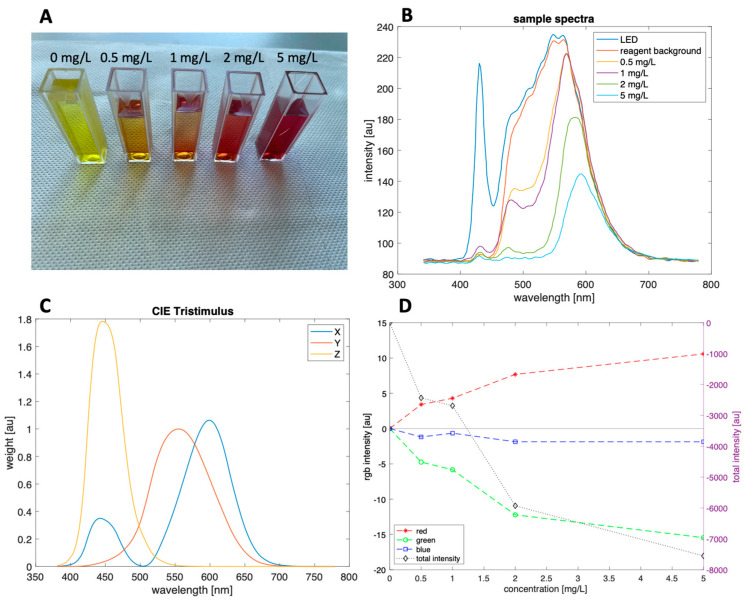
Spectrophotometric characterization of the Griess reaction in the microfluidic chip. (**A**): Griess reaction products at different nitrites concentration. (**B**): spectra collected by the C12666MA spectrophotometer. The white LED spectrum is labelled as water and corresponds to the chip filled with tap water. (**C**): spectral behaviour of the weights of the CIE colour functions. (**D**): calculated red, green, and blue signals normalized respect to the value at 0 mg/L. In this plot also the variation of the total intensity of light is also shown.

Spectra have been acquired at different concentrations of nitrites obtained spiking NaNO_2_ in distilled water. Figure 3A shows the Griess reaction products at growing concentrations of nitrites. The cuvette labeled as 0 mg/L corresponds to the intrinsic color of the reagents. Figure 3B shows the collected spectra. In the case of pure water, the collected spectrum shows the typical features of a white LED. The addition of reagents and the reaction with nitrites result in a progressive decrease of the intensity of spectrum in the blue and green spectral regions.

To evaluate the feasibility of color sensors detection, colors have been estimated from the spectra. For the scope, the CIE color matching functions (*x_λ_*, *y_λ_*, *z_λ_*) have been considered [27]. These functions define the contribution of each wavelength to the CIE tristimulus values: *X*, *Y* and *Z*. Figure 3C show the spectral weights of the three values.

The CIE tristimulus parameters are calculated integrating the spectra (*P_λ_*) weighted by the corresponding CIE color function:

$$X=\int_{380}^{780}x_{\lambda }\cdot P_{\lambda }\;d\lambda ;\;Y=\int_{380}^{780}y_{\lambda }\cdot P_{\lambda }\;d\lambda ;Z=\int_{380}^{780}z_{\lambda }\cdot P_{\lambda }\;d\lambda ;\;$$

The tristimulus parameters have been converted into RGB values using the MATLAB function *xyz2rgb*. Additionally, the total intensity of the spectra, corresponding to the gray level intensity of light, have also been calculated:

$$\;I=\int_{380}^{780}P_{\lambda }\;d\lambda$$

The spectral analysis shows the feasibility of Griess reaction detection occurring inside the microfluidic chip. Furthermore, the evaluation of color parameters suggests that color detection retains the spectral changes in the reaction products. This result makes possible the integration of more simple and less expensive and power consuming detectors.

### 3.3. Colorimetric Detection and Deployable System

The spectrophotometer was replaced by an integrated color sensor made of four photodiodes. Three photodiodes are coupled with color filters (red, green, and blue) while the fourth detects the whole spectra and thus it provides the grayscale intensity. An infrared filter, common to all photodiodes, removes the wavelengths larger than 700 nm confining the detection to the visible light. The device provides a 16-bit output for each photodiode. Similarly, to the spectrophotometer, a white LED, integrated into the sensor breakout board, provides illumination for color detection.

The reagents and water inputs were controlled by piezoelectric micropumps that ensure the constant delivery of samples and reagents in each measurement. The chip is designed to consume a total of 1ml of sample and reagent for each measurement. The whole system is controlled by an Arduino nano board programmed with custom firmware. An output RS232 connection is available to connect the system with other sensors or data aggregators (IADAS—Integrated Autonomous Data Acquisition System).

**Figure 4:**
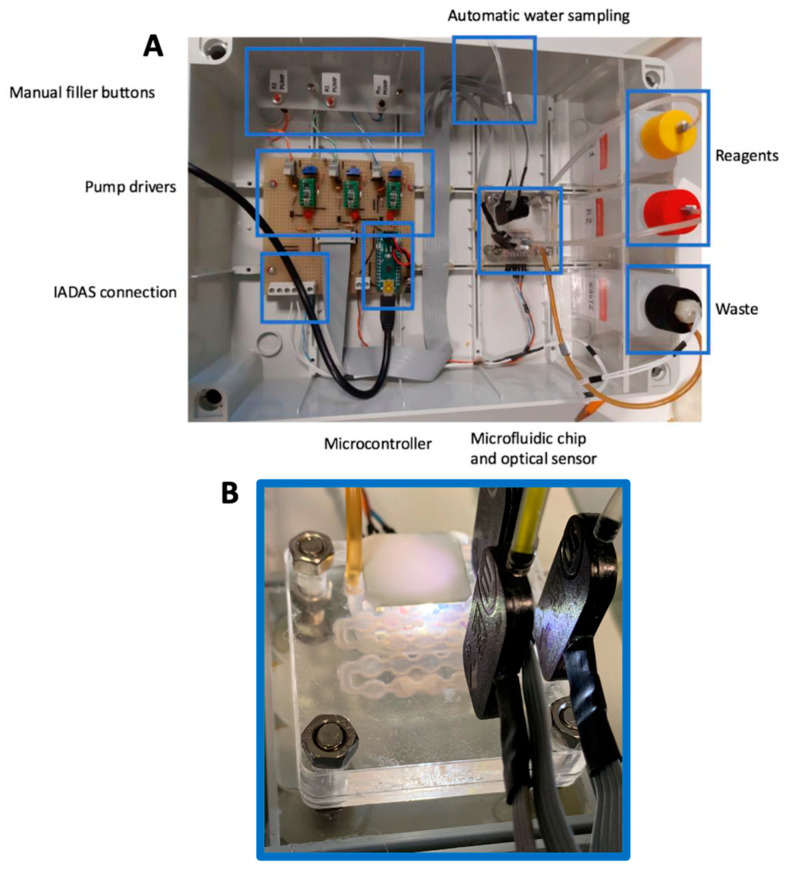
(**A**): top view of the sensor platform with evidenced subsystems. (**B**): Close view of the lab-on-a-chip connected to pumps and optoelectronics components.

The sensor platform is complemented by a reagents reservoir of 15 mL each and a container for wastes. This configuration allows over 400 measurements before reagents refill while the total measurement time. From sample uptake to lab-on-a-chip discharge, is less than 120 s. Reagents and wastes levels are controlled by water level sensors that send an alarm in case of filling of wastes and/or emptying of reagents. Figure 4 shows the total view of the sensor system assembled inside an industrial box IP68.

### 3.4. Colorimetric Sensor Calibration

The nitrites concentration has been investigated in the range 0.1–15 mg/L. Measurements were repeated four times in random sequences. The grayscale intensity signal of the detector has been used to normalize the signals from red, green and blue channels. In practice, the signal of each color channel has been divided by the signal of the gray level channel. The normalized signals have been rescaled in 255 levels in order to remove noisy and fluctuating values. The sensor response to a given sample has been obtained as the difference between the sensor signal in the sample and the sensor signal at reference corresponding to the reagent color before the addition of the water sample.

**Figure 5:**
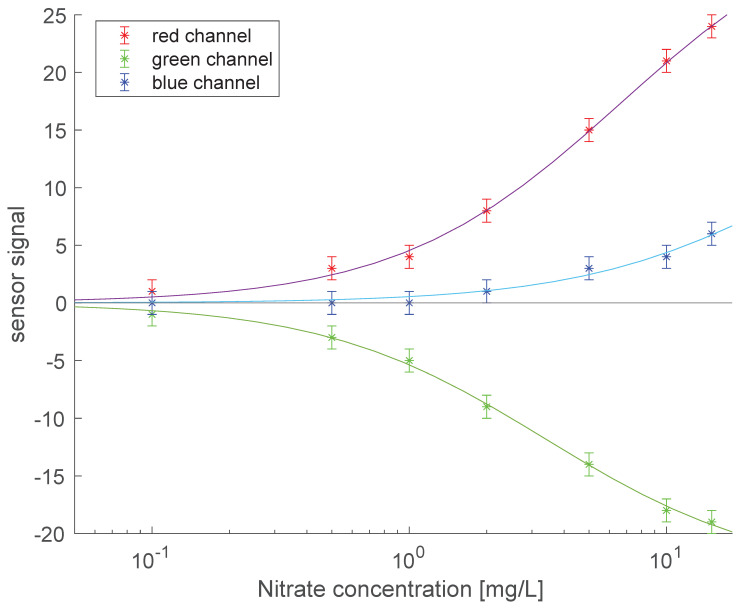
Colorimetric sensor calibration curves.

Figure 5 shows the calibration curves. The curves are in good agreement with those calculated from the measured spectra applying theoretical color functions. In practice, the actual filters implemented in the devices may deviate from those predicted by the CIE color functions. Furthermore, the optical arrangement of the colorimetric sensor is different from the spectrophotometric. Differences in aperture angles and light source positions may be the source of changes in light intensity.

As expected from Figure 3D, red and green channels are almost equivalent to describe the concentration of nitrites while the contribution of the blue channels is practically negligible. The sensor response fits well with a Michaelis–Menten function:

$$y=y_{max}\frac{x}{c_{0}+x}$$

where *y* is the signal and x is the concentration, *y_max_* is the larger estimated signal and *c*_0_ is the concentration at which half of the maximum signal is achieved. Table 1 lists the results of the fit performed in Matlab using the cftool gui, the Levenberg–Marquardt algorithm was used.

In the Griess assay, the relationship between absorbance and nitrite concentration is known to be linear [17]. Thus, the good results of the fit with the Michaelis–Menten equation do not imply that the Griess reaction is characterized by enzyme-like dynamics, rather it has to be considered as the consequence of the non-linearity of the detector and the non-linear effects in light scattering when the intensity of reflected light becomes low.

The analytical function between signal and concentration enables to estimate the minimum detectable concentration (MDC) calculated as the ratio of the measurement error and the sensitivity at the origin [28]. Here we can consider the change of 1 unit of sensor signal as the minimum detectable signal, while the sensitivity is the derivative of the Michaelis–Menten equation at the origin. Thus, MDC is evaluated as:

$$c_{MDC}=\frac{\Delta y_{err}}{S_{c=0}}=\frac{\Delta y_{err}}{{\frac{dy}{dx}}_{c=0}}=\frac{\Delta y_{err}}{\frac{y_{max}}{c_{0}}}$$

MDC is shown in Table 1. Both red and green channel achieve an MDC of 0.1 mg/L while more than 10 time larger is the MDC achieved using the signals of the blue channel.

## 4. Conclusions

Microfluidics enables the automatization of analytical analysis giving the possibility to exploit well-known and reliable assays. Due to the limited amount of reagents, such a system can perform a large number of repeated measurements providing a long time monitoring of analytes. The detection of reaction products, and then the quantification of analytes can be provided by sensors embedded in the microfluidic device. Optical detection is particularly efficient from the point of view of the integration because it only requires optical access to the reaction product. This means from one side that the lab-on-a-chip has to be made with a transparent material and that the optical path has to be less disturbed as possible. One of the main drawbacks of these systems is the generation of air bubbles during the liquid mix. The specific design is necessary to ensure a homogeneous mix and the avoidance of bubbles during the sample displacement along the channels [18,29].

In this paper, we presented a lab-on-a-chip platform for the automatization of the Griess reaction for the detection of nitrites in water. The platform has been developed to monitor nitrites in an aquaculture farm. Colorimetric detection has been performed with integrated color sensors that can capture the color changes due to nitrites concentration above 0.1 mg/L. The achieved MDC corresponds to about 2 µM, such a value is sufficient for aquaculture purposes, however this is larger respect to literature values. The limit of detection of Griess reaction can reach 0.02 µM in a lab-on-chip [22] and 1 µM immobilized on paper [25]. Such a difference can be explained considering the limited properties of the low-cost optoelectronic devices and in the use of a commercial nitrite kit specific for nitrites detection for fish welfare. Indeed both the examples reported above are based on modified Griess reagents formulation.

Furthermore, this result has been obtained with a optical path of about 1 mm (see Figure 1).The increase of optical path, achieved deviating the optical rays parallel to the chip, has been found to strongly decrese the minimum detectable concentrations [30]. Although its wide use, Griess reaction is affected by limited selectivity respect for instance to ionic species, the presence of pigments and anti-oxidants [11]. However, in this paper we have been mostly interested to test the platform respect to a colorimetric assay. This general approach of the platform design allows the use of the system with any other colorimetric assay for different analytes and different substrates. Future work will include the improvement of chip design to increase the mixing efficiency in order to reduce the amount of reagent per analysis [31], and the integration of optical components [32]. Furthermore, the use of the platform will be extended to other analytes of interest in aquaculture, for instance incorporating molybdenum assays for phosphate detection [33].

## Figures and Tables

**Table 1:** Parameters of best fit of sensor responses with the Michaelis–Menten equation, the errors are calculated with 95% of confidence bound.

Channel	Ymax	C_0_ [mg/L]	R^2^	MDC [mg/L]
red	34 ± 3	6 ± 1	0.998	0.1
green	−23 ± 2	3 ± 1	0.998	0.1
blue	20 ± 3	35 ± 5	0.978	1.7
